# Possible Tomonaga-Luttinger spin liquid state in the spin-1/2 inequilateral diamond-chain compound K_3_Cu_3_AlO_2_(SO_4_)_4_

**DOI:** 10.1038/s41598-017-16935-9

**Published:** 2017-12-01

**Authors:** Masayoshi Fujihala, Hiroko Koorikawa, Setsuo Mitsuda, Katsuhiro Morita, Takami Tohyama, Keisuke Tomiyasu, Akihiro Koda, Hirotaka Okabe, Shinichi Itoh, Tetsuya Yokoo, Soshi Ibuka, Makoto Tadokoro, Masaki Itoh, Hajime Sagayama, Reiji Kumai, Youichi Murakami

**Affiliations:** 1 Tokyo University of Science, Department of Physics, Tokyo, 162-8601 Japan; 2 Tokyo University of Science, Department of Applied Physics, Tokyo, 125-8585 Japan; 3 Tohoku University, Department of Physics, Sendai, 980-8578 Japan; 4 High Energy Accelerator Research Organization, Muon Science Laboratory and Condensed Matter Research Center, Institute of Materials Structure Science, Tsukuba, 305-0801 Japan; 5 High Energy Accelerator Research Organization, Neutron Science Division, Institute of Materials Structure Science, Tsukuba, 305-0801 Japan; 6 Tokyo University of Science, Department of Chemistry, Tokyo, 162-8601 Japan; 7 High Energy Accelerator Research Organization, Photon Factory, Institute of Materials Structure Science, Tsukuba, 305-0801 Japan

## Abstract

K_3_Cu_3_AlO_2_(SO_4_)_4_ is a highly one-dimensional spin-1/2 inequilateral diamond-chain antiferromagnet. Spinon continuum and spin-singlet dimer excitations are observed in the inelastic neutron scattering spectra, which is in excellent agreement with a theoretical prediction: a dimer-monomer composite structure, where the dimer is caused by strong antiferromagnetic (AFM) coupling and the monomer forms an almost isolated quantum AFM chain controlling low-energy excitations. Moreover, muon spin rotation/relaxation spectroscopy shows no long-range ordering down to 90 mK, which is roughly three orders of magnitude lower than the exchange interaction of the quantum AFM chain. K_3_Cu_3_AlO_2_(SO_4_)_4_ is, thus, regarded as a compound that exhibits a Tomonaga-Luttinger spin liquid behavior at low temperatures close to the ground state.

## Introduction

Identifying spin liquid phases in the ground state is one of hot topics in the field of low-dimensional quantum magnets. Three-dimensional orders induced by magnetic interactions between one-dimensional (1D) chains/two-dimensional layers, however, prevent the spin-liquid ground state. It is thus essential to search for quantum magnets that possess negligibly weak inter chain/layer interactions.

One of possible spin liquids in 1D system is a Tomonaga-Luttinger (TL) spin liquid, where spin-spin correlation decays algebraically with distance. Azurite Cu_3_(CO_3_)_2_(OH)_2_ that contains spin-1/2 distorted diamond chains^1–4^ is a possible candidate for the TL spin liquid, since the ground state of the distorted diamond-chain is expected to belong to an alternating dimer-monomer phase where neighboring monomers are connected via the dimer in between and an effective Heisenberg 1D chain controls low-energy excitations^5,6^. In fact, a recent theoretical approach based on density functional theory together with numerical many-body calculations has proposed a microscopic model for the azurite, which includes two energy scales coming from singlet dimer and 1D Heisenberg chain^7^. Although the model predicts the TL spin liquid, three-dimensional magnetic interactions in the azurite cause the magnetic order at 1.85 K.

**Figure 1:**
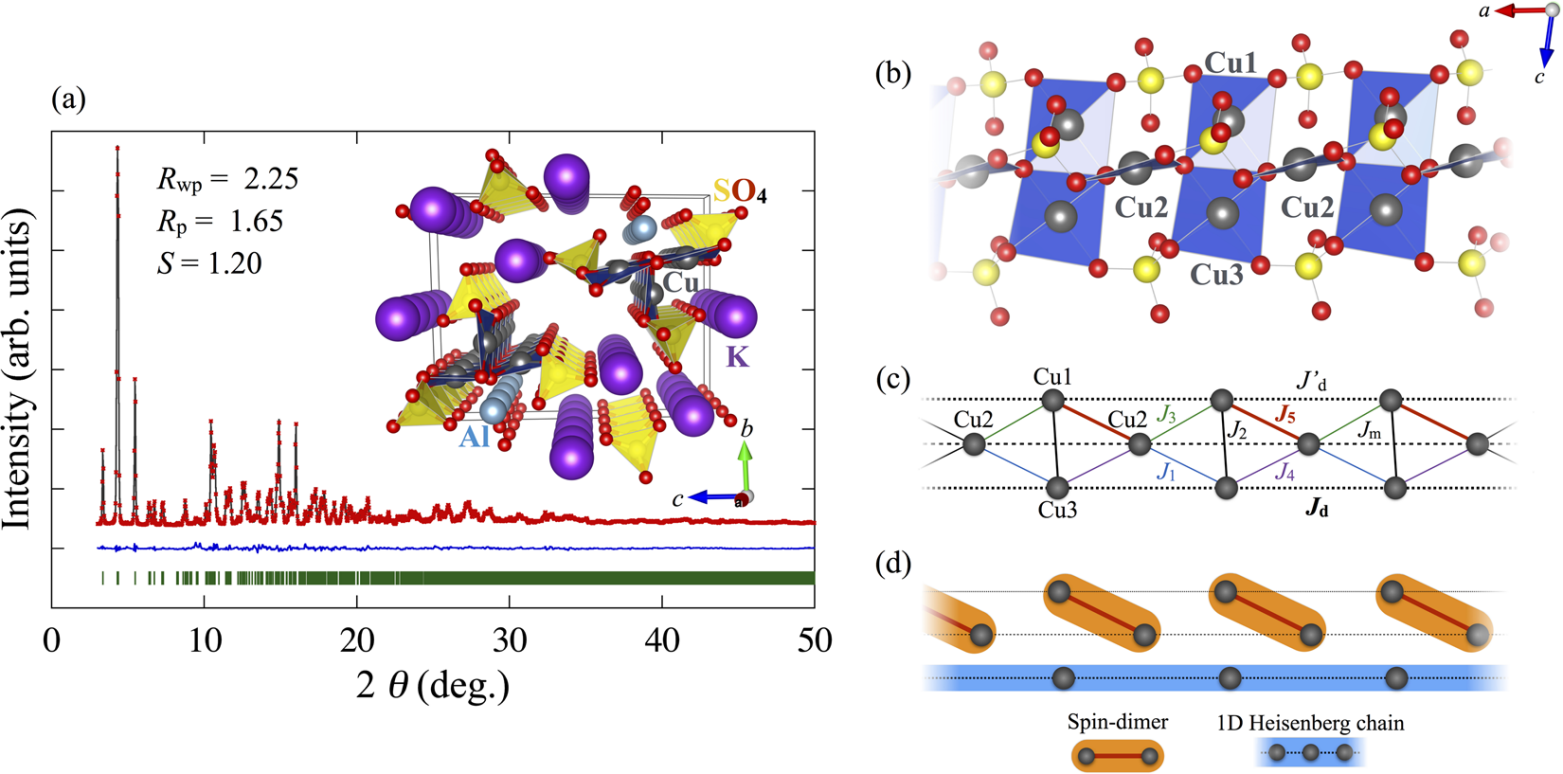
(**a**) Synchrotron XRD intensity pattern (filled red circles) observed for K_3_Cu_3_AlO_2_(SO_4_)_4_ at room temperature, the result of Rietveld refinement using the computer program RIETAN-FP^22^ (black solid line), and difference between the calculated and observed intensities (blue solid line). The green vertical bars indicate the position of Bragg reflection peaks. The inset shows the crystal structure of K_3_Cu_3_AlO_2_(SO_4_)_4_ featuring a large inter-chain spacing. (**b**) The diamond chain of K_3_Cu_3_AlO_2_(SO_4_)_4_, which consists of Cu^2+^ ions (grey spheres) along the *a*-axis with nearby oxygen (red spheres) and sulfur ions (yellow spheres). (**c**) Effective spin model of K_3_Cu_3_AlO_2_(SO_4_)_4_ with the nearest-neighbor exchange couplings *J*_*i*_ (*i* = 1 to 5), and the next nearest-neighbor exchange couplings of *J*_m_, *J*_d_, and ${J}_{{\rm{d}}}^{\prime}$. (**d**) Spin configuration of the ground state for K_3_Cu_3_AlO_2_(SO_4_)_4_.

Recently a highly 1D inequilateral diamond-chain compound alumoklyuchevskite K_3_Cu_3_AlO_2_(SO_4_)_4_ has been reported by some of the present authors^8^. There is no long-range magnetic order down to at least 0.5 K as evidenced by specific heat measurements. The magnetic susceptibility exhibits a double broad peak at around 200 K and 50 K. By analyzing the magnetic susceptibility, an effective model for K_3_Cu_3_AlO_2_(SO_4_)_4_ has been proposed^9^, where the dimer is formed by one of the four sides in the diamond and the remaining spins form a 1D Heisenberg chain, as shown in Fig. 1(d). The model is different from that for the azurite.

In this paper, we present detailed studies of K_3_Cu_3_AlO_2_(SO_4_)_4_ through single-crystal and powder X-ray diffraction (XRD), inelastic neutron scattering (INS), and muon spin rotation/relaxation (*μ*SR) spectroscopy. These experimental results indicate that K_3_Cu_3_AlO_2_(SO_4_)_4_ is an appropriate model material for the investigation of the TL spin liquid state at low temperatures close to the ground state.

## Results and Discussion

### Crystal structure refinement

The Cu-O-Cu angle significantly influences on the value of the exchange interactions, the variation of the angles can give strong bond-dependent exchange interactions^10^. Therefore, the crystal structure refinement of K_3_Cu_3_AlO_2_(SO_4_)_4_ is necessary to determine the magnetic exchange interactions. The space group of the mineral alumoklyuchevskite K_3_Cu_3_Al_0.64_Fe_0.36_O_2_(SO_4_)_4_, is *C*2, as reported by Krivovichev *et al*.^11^. However, the previous powder XRD data showed that it is not consistent with synthesized K_3_Cu_3_AlO_2_(SO_4_)_4_.

The space group and structural parameters for the synthesized material are determined from single crystal XRD and synchrotron powder XRD to be $P\bar{1}$ and *a* = 4.9338(5) Å, *b* = 11.923(5) Å, and *c* = 14.578(5) Å, with *α* = 87.309(5)°, *β* = 80.837(3)°, and *γ* = 78.458(6)°, respectively. As shown by Rietveld refinement results, there is no impurity peaks [Fig. 1(a)]. The inset of Fig. 1(a,b) show that K_3_Cu_3_AlO_2_(SO_4_)_4_ contains magnetic Cu^2+^ ions in an inequilateral diamond-chain arrangement along the *a*-axis direction. The nearest-neighbor magnetic couplings, *J*_*i*_ (*i* = 1 to 5), are the superexchange interactions through Cu-O-Cu bonds: *J*_1_ (*J*_4_) through Cu2-O-Cu3 bond with bond angle 101.59(17)° (105.38(18)°), *J*_2_ through Cu1-O-Cu3 with two bond angles 96.6(2)° and 96.7(2)°, and *J*_3_ (*J*_5_) through Cu1-O-Cu2 with 104.51(18)° (127.8(2)°), see Fig. 1(c). In addition, the exchange interactions through the Cu-O-S-O-Cu exchange paths are denoted by *J*_m_, *J*_d_, ${J}_{{\rm{d}}}^{\prime}$ in Fig. 1(b). *J*_5_ with the largest angle is expected to be the largest antiferromagnetic (AFM) interaction, while *J*_2_ with the smallest angle is considered to be a ferromagnetic (FM) interaction^10^.

The values of the exchange interactions have been obtained by fitting the temperature dependence of the magnetic susceptibility with calculated one^9^: *J*_1_ = *J*_3_ = *J*_4_ = −30 K, *J*_2_ = −300 K, *J*_5_ = 510 K, and ${J}_{{\rm{m}}}={J}_{{\rm{d}}}={J}_{{\rm{d}}}^{\prime} =75$ K. These values are completely different from those of azurite^7^: the *J*_2_ bond, where the singlet dimer is located in azurite, is FM, while the singlet dimer and a 1D chain is formed on the *J*_5_ bond and *J*_d_ bond, respectively, as shown in Fig. 1(c,d). The alternating dimer-monomer model realized in azurite is, thus, not the case in K_3_Cu_3_AlO_2_(SO_4_)_4_. The TL spin liquid in this compound is formed along the *J*_d_ bond. We will show below that our experimental results are in excellent agreement with this prediction.

### Spinon continuum and spin-singlet dimer excitations in the inelastic neutron scattering spectra

The theoretical study predicted that a gapless low-energy spin excitation and gap excitation are observed by INS experiment, because the spin dimer together with a nearly isolated 1D Heisenberg spin chain characterizes magnetic properties of K_3_Cu_3_AlO_2_(SO_4_)_4_^9^.

**Figure 2:**
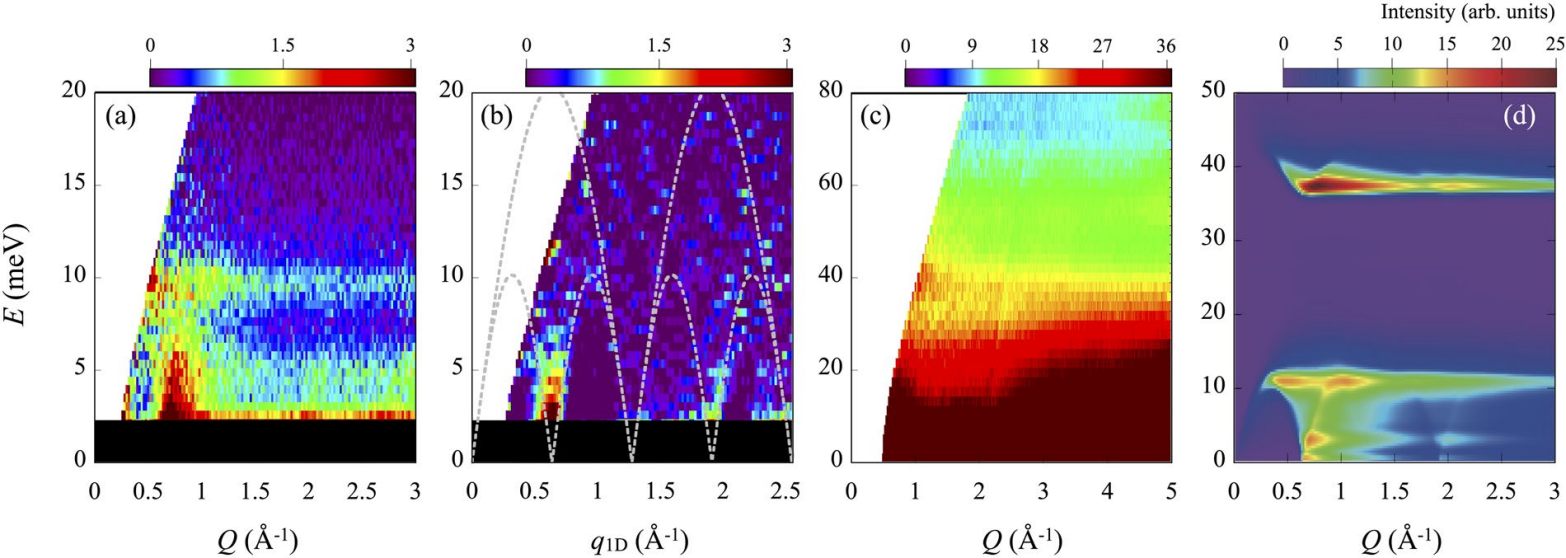
INS spectra for K_3_Cu_3_AlO_2_(SO_4_)_4_. (**a**) Magnetic scattering contribution, extracted the 100 K data from the 4 K data corrected for the phonon population factor (*E*_*i*_ = 45.95 meV). (**b**) Single-crystal-like data obtained by applying the conversion method. The superimposed gray dashed lines indicate the lower and upper energy boundaries of the continuum given by (*πJ*_d_/2)|sin(*Qa*)| and *πJ*_d_|sin(*Qa*/2)|^13^, respectively, where *a* is the lattice parameter in the chain direction. (**c**) Experimental raw data measured at 4 K with incident neutron energy of 205.8 meV. (**d**) Simulated powder-averaged INS spectrum.

INS experiments are performed on the powder samples using the high resolution chopper spectrometer HRC at MLF of J-PARC. Figure 2(a) shows the magnetic scattering contribution at 4 K, which is obtained by subtracting the phonon contribution from the observed spectrum at 100 K (see Supplementary Information Sec. I). The strong flat signal is seen at approximately 10 meV, indicating that there is the van Hove singularity of spinon continuum edges at this energy. On the other hand, especially in the 1D system, powder orientational average obfuscates the INS intensity distribution and displaces the *Q* position from the true *q*_1D_ position considerably, where *Q* denotes the experimental wavenumber in powder experiment and *q*_1D_ is defined as the wavenumber along 1 chain direction. Thus, we also use a conversion method mitigating the powder average effect for a one-dimensional system^12^. This method enhances the vertically rising part of dispersion and corrects the *Q* position to the corresponding *q*_1D_ position on the basis of the differential with respect to *Q*. The converted result is shown in Fig. 2(b). The spinon continuum edges rise up from the Brillouin zone centers in chain direction, *q*_1D_ = *π*/*a* = 0.64 Å^−1^ and 3*π*/*a* = 1.91 Å^−1^. In addition, the observed lower-energy boundary of the continuum is consistent with the theoretical line for the 1D spin-1/2 Heisenberg antiferromagnet given by (*πJ*_d_/2)|sin(*Qa*)|^13^, indicating that the velocity of spin excitations of K_3_Cu_3_AlO_2_(SO_4_)_4_ is an appropriate for the 1D spin-1/2 Heisenberg antiferromagnet.

Figure 2(c) shows the data measured with *E*_*i*_ = 205.8 meV. A signal is observed at around *E* = 40 meV and *Q* = 1.0 Å^−1^. The signal due to magnetic excitations is generally enhanced at low-*Q* values, whereas phonon excitations are dominant at high-*Q*. Therefore, we consider that this signal comes from magnetic excitations.

The dynamical spin structure factor for the proposed model has been calculated by the dynamical density-matrix renormalization group^9^. In order to compare the calculated spectrum with the powder INS spectra in Fig. 2(a,c), we simulate the powder-averaged INS spectrum including the magnetic form factor of Cu^2+^ by using a conversion technique (see Eq. (2) of ref.^12^). Figure 2(d) shows the simulated powder-averaged INS spectrum. The agreement with experimental data in Fig. 2(a,c) is fairly good. Therefore, we are confident that the proposed exchange interactions and the spin configuration shown in Fig. 1(c) are appropriate for this compound. In other words, the low-energy excitation is characterized by a TL spin liquid.

### *μ*SR evidence for a quantum spin liquid state in K_3_Cu_3_AlO_2_(SO_4_)_4_

Quantum spin fluctuations in K_3_Cu_3_AlO_2_(SO_4_)_4_ are investigated by using zero-field (ZF) and longitudinal-field (LF) *μ*SR measurements for a powder sample in the temperature range from 90 mK to 300 K at MLF of J-PARC.

**Figure 3:**
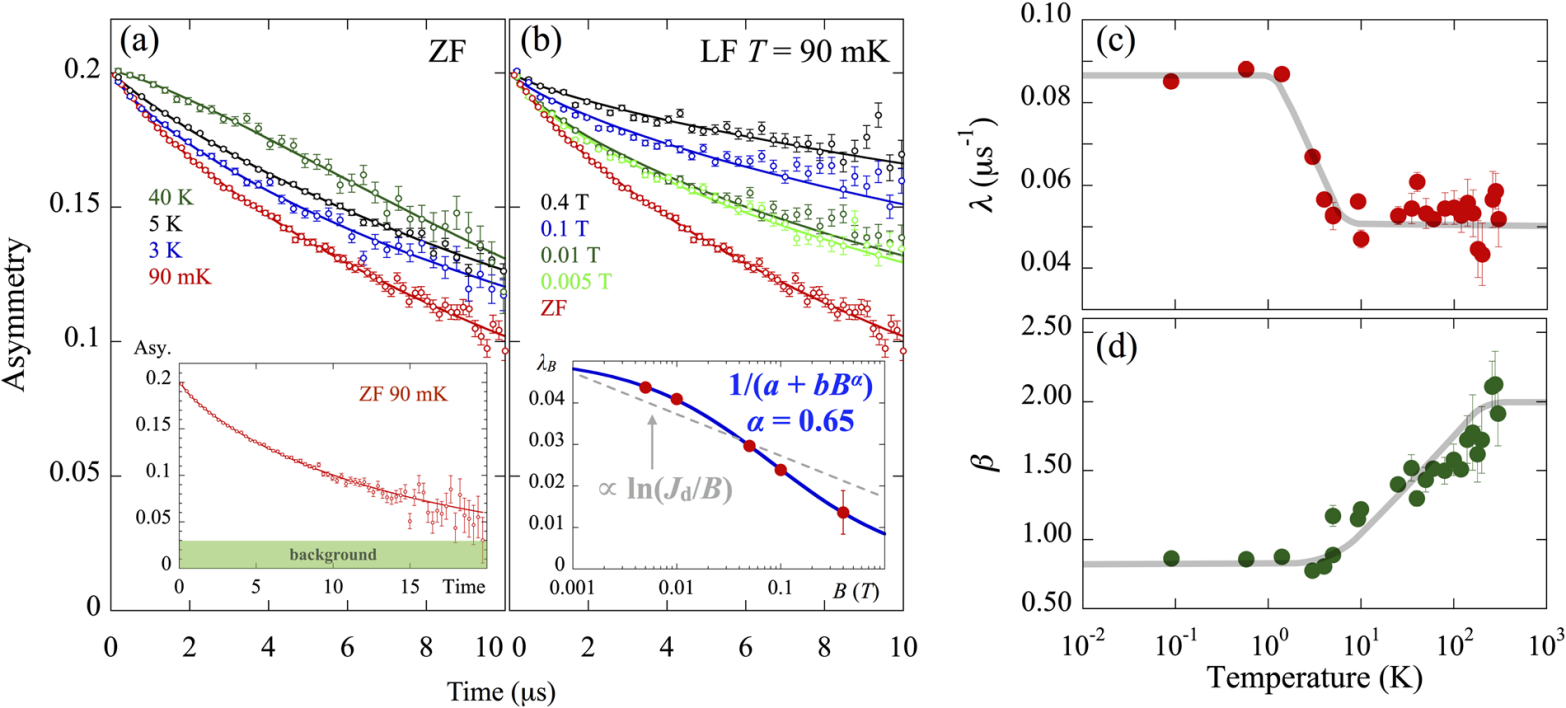
(**a**) ZF-*μ*SR spectra (using a dilution refrigerator) at representative temperatures (see Supplementary Information Sec. II for the spectra obtained using ^4^He cryostat). The thick lines behind the data points are curves fitted by using the stretched exponential function (see text). The inset shows the ZF-*μ*SR spectrum measured at 90 mK, which decreases continuously without oscillations up to 20 *μ*s. (**b**) *μ*SR spectra measured at 90 mK under ZF and representative longitudinal magnetic fields. The inset shows the magnetic field dependence of the muon spin relaxation rate *λ*_*B*_. The blue solid line and gray dashed line are curves fitted by using the power-law and logarithmic equation (see text). (**c**) Temperature dependence of the muon spin relaxation rate *λ*. (**d**) Temperature dependence of the stretching exponent *β*. The gray solid lines in (**c**,**d**) are the guide to eyes.

The ZF-*μ*SR spectra are fitted by the stretched exponential function $a(t)={a}_{1}\,\exp [-{(\lambda t)}^{\beta }]+{a}_{{\rm{BG}}}$, where *a*_1_ is an intrinsic asymmetry *a*_1_ = 0.17, *a*_BG_ is a constant background *a*_BG_ = 0.031, *λ* is the muon spin relaxation rate, and *β* is the stretching exponent. The spectra at representative temperatures are presented in Fig. 3(a). The combined effect of these multiple nuclear dipole fields leads to a phenomenologically described relaxation function of the stretched exponential. The field distribution and the stretching exponent at high temperatures are approximately given by Δ_nuclear_ = *λ*/*γ*_*μ*_ = 0.6 G and *β* ≈ 2, respectively, which are typical for a nuclear dipole field. The ZF spectrum at the lowest temperature 90 mK decreases continuously without oscillations up to 20 *μ*s (see the inset of Fig. 3(a)). If this ZF spectrum is due to static magnetism, the internal field should be approximately 1.1 G. However, the relaxation is clearly observed, even in the LF at 0.4 T, which is evidence for the fluctuation of Cu^2+^ electron spins without static ordering down to 90 mK.

The LF spectra measured at 90 mK are also fitted by a stretched exponential function, where the stretching exponent is fixed as *β* = 0.748. Using the power-law represented by 1/(*a* + *bH* ^*α*^)^14,15^ with an unconventional value of *α* = 0.65, where *a* and *b* are dependent on the fluctuation rate and fluctuating field, we obtain a good fitting to the magnetic field dependence of the muon spin relaxation rate *λ*_*B*_, as shown in the inset of Fig. 3(b). Incidentally, the 1/(*a* + *bH* ^2^) is a standard case that the *λ*_*B*_ obeys the Redfield equation. The *λ*_*B*_ for ballistic spin transport in the 1D spin-1/2 Heisenberg antiferromagnet follows a logarithmic relation *λ*_*B*_ ∝ ln(*J*/*B*). In contrast, for diffusive transport, the *λ*_*B*_ obeys the *B* ^−0.5^ power-law^16,17^. As shown in the inset of Fig. 3(b), the line given by ln(*J*_d_/*B*) is clearly a poor description of the data. Although *α* is a little higher than the ideal value 0.5, it is broadly consistent with a 1D diffusive picture of the spin fluctuations.

The abrupt increase of the relaxation rate *λ* below 1.5 K and the gradual decrease of *β* are observed, as shown in Fig. 3(c,d), respectively. Although such behaviors are not expected in the TLL spin-liquid system, they have been often seen in other spin-liquid candidates. In these systems, the increase of at low temperatures was ascribed to unpaired spins, which originate from chain breaks^18,19^. The presence of unpaired spins in K_3_Cu_3_AlO_2_(SO_4_)_4_ is evident in our previous study^8^.

All of these *μ*SR results strongly support the formation of a quantum spin liquid at very low temperature in K_3_Cu_3_AlO_2_(SO_4_)_4_.

## Conclusion

In summary, a spin-1/2 inequilateral diamond-chain compound K_3_Cu_3_AlO_2_(SO_4_)_4_ has been experimentally examined by single-crystal and powder X-ray diffraction, muon spin rotation/relaxation spectroscopy, and inelastic neutron scattering. By comparing the INS experimental data with calculations for a theoretically proposed model, we have confirmed that the compound is described by a composite structure consisting of singlet dimers and a one-dimensional Heisenberg chain, which is different from an alternating dimer-monomer model corresponding to azurite. Since the low-energy excitations are described by the one-dimensional Heisenberg model and there is no three-dimensional long-range order down to 90 mK, K_3_Cu_3_AlO_2_(SO_4_)_4_ is regarded as a typical compound that exhibits a TL spin liquid behavior at low temperatures close to the ground state. K_3_Cu_3_AlO_2_(SO_4_)_4_ would further contribute to experimental efforts in uncovering exotic properties of the TL spin liquid, such as spinon spin currents^20^ and ballistic thermal conduction^21^.

## Methods

Single phase polycrystalline K_3_Cu_3_AlO_2_(SO_4_)_4_ was synthesized by solid-state reaction in which high-purity K_2_SO_4_, CuO, CuSO_4_, and AlK(SO_4_)2 powders were mixed in a molar ratio of 1:2:1:1, followed by heating at 600 °C for three days and slow cooling in air. Significant efforts were made to grow single crystals, whereby several tiny single crystals of K_3_Cu_3_AlO_2_(SO_4_)_4_ were successfully grown by heating the preliminarily grown powder at temperatures as high as 600° in a sealed and evacuated quartz tube for one week with slow cooling. Single crystal XRD data were collected on a Bruker AXS Smart ApexII ULTRA CCD diffractometer using MoK*α* (*λ* = 0.71073 Å) radiation. Synchrotron powder XRD data were collected using an imaging plate diffractometer installed at BL-8B of the Photon Factory. An incident synchrotron X-ray energy of 18.0 keV (0.68892 Å) was selected. The INS experiments are performed on the HRC, installed at BL12 beamline at the Materials and Life Science Experimental Facility (MLF) of the Japan Proton Accelerator Research Complex (J-PARC). At the HRC, white neutrons are monochromatized by a Fermi chopper synchronized with the production timing of the pulsed neutrons. The energy transfer was determined from the time-of-flight of the scattered neutrons detected at position sensitive detectors. A 400 Hz Fermi chopper was used to obtain a high neutron flux. A GM-type closed cycle cryostat was used to achieve 100 K and 4 K. The energies of incident neutrons were *E*_*i*_ = 205.8 meV and 45.95 meV (second frame), which yielded an energy resolutions of *E* = 5 and 1 meV at the elastic position. ZF and LF *μ*SR experiments are performed using the spin-polarized pulsed surface-muon (*μ* ^+^) beam at the D1 beamline of the MLF of J-PARC. The spectra were collected in the temperature range from 90 mK to 300 K using a dilution refrigerator and ^4^He cryostat.

## Electronic supplementary material

Supplementary Information
