# Supplementary material for: Possible Tomonaga-Luttinger spin liquid state in the spin-1/2 inequilateral diamond-chain compound K3Cu3AlO2(SO4)4
Source: Sci Rep. 2017 Dec 1;7:16785. doi: 10.1038/s41598-017-16935-9 (PMC5711793; doi:10.1038/s41598-017-16935-9)
Supplement: Supplementary file 1 — Supplementary Information [file 41598_2017_16935_MOESM1_ESM.pdf]

Possible Tomonaga-Luttinger spin liquid state in the spin-1/2 inequilateral diamond-chain compound $K_3Cu_3AlO_2(SO_4)_4$

Masayoshi Fujihala[1,*], Hiroko Koorikawa[1], Setsuo Mitsuda[1], Katsuhiro Morita[2], Takami Tohyama[2], Keisuke Tomiyasu[3], Akihiro Koda[4], Hirotaka Okabe[4], Shinichi Itoh[5], Tetsuya Yokoo[5], Soshi Ibuka[5], Makoto Tadokoro[5], Masaki Itoh[5], Hajime Sagayama[5], Reiji Kumai[5], and Youichi Murakami[5]

[1]Tokyo University of Science, Department of Physics, Tokyo, 162-8601, Japan
[2]Tokyo University of Science, Department of Applied Physics, Tokyo, 125-8585, Japan
[3]Tohoku University, Department of Physics, Sendai, 980-8578, Japan
[4]High Energy Accelerator Research Organization, Muon Science Laboratory and Condensed Matter Research Center, Institute of Materials Structure Science, Tsukuba, 305-0801, Japan
[5]High Energy Accelerator Research Organization, Neutron Science Division, Institute of Materials Structure Science, Tsukuba, 305-0801, Japan
[6]Tokyo University of Science, Department of Chemistry, Tokyo, 162-8601, Japan
[7]High Energy Accelerator Research Organization, Photon Factory, Institute of Materials Structure Science, Tsukuba, 305-0801, Japan
*fujihara@nsmsmac4.ph.kagu.tus.ac.jp

Supplementary Information

I. INS EXPERIMENT

To obtain information on the magnetic excitation along the chain direction from the powder INS spectrum, the conversion method developed by Tomiyasu *et al.*[1] was used. Figures S1(a) and (b) show the spectra measured at 4 K and 100 K, respectively. The magnetic scattering contribution at 4 K (Fig. S1(c)) is obtained by subtracting the phonon contribution from the observed spectrum. The 100 K data is used to determine the phonon contribution at 4 K by dividing the phonon signal by the phonon thermal population factor at 100 K and multiplying by the population factor at 4 K.

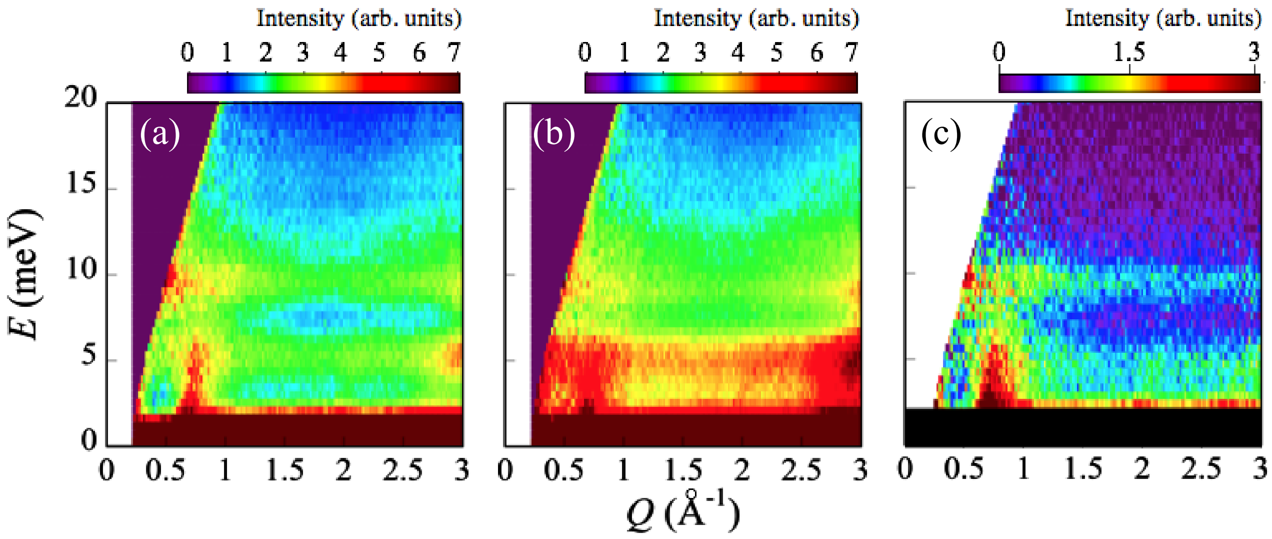

Figure S1. Experimental INS spectra for $K_3Cu_3AlO_2(SO_4)_4$ with an incident neutron energy 45.95 meV measured at (a) 4 K and (b) 100 K. (c) Magnetic scattering contribution at 4 K.

II. μSR EXPERIMENT

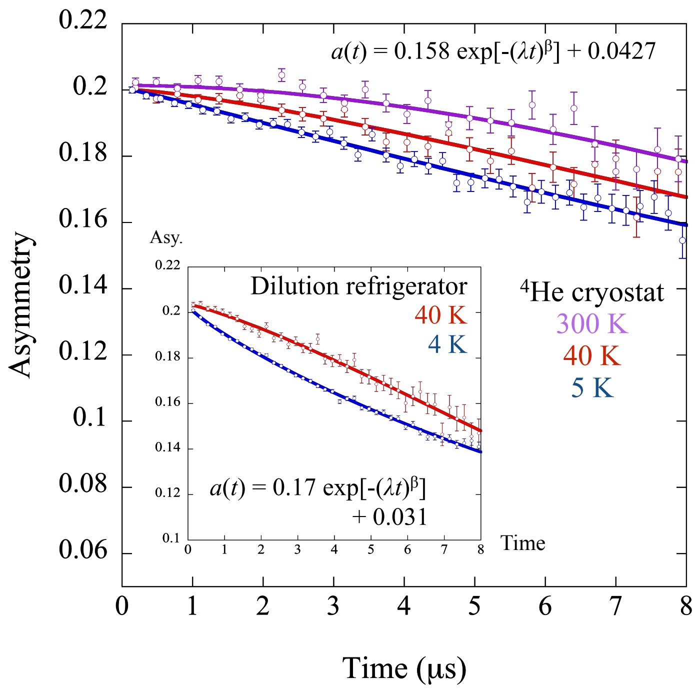

Figure S2. ZF-μSR spectra (using a ^{4}He cryostat) at representative temperatures. The thick lines behind the data are fitted curves as described in the text. The inset plot shows the ZF-μSR spectra measured at temperatures of 40 K and 5 K achieved using a dilution refrigerator.

The spectra were collected in the temperature range from 90 mK to 300 K using a dilution refrigerator and ^{4}He cryostat. Figure S2 shows the ZF-μSR spectra that were obtained using a ^{4}He cryostat. All spectra are fitted by the stretched exponential function $a(t) = a_1 \exp[-(\lambda t)^{\beta}] + a_{BG}$. Here, a_1 is an intrinsic asymmetry and a_{BG} is a constant background that represents muons that missed the sample; therefore, the initial asymmetry is the sum of a_1 and a_{BG}. a_{BG} is determined by the size, shape and density of the sample and the sample environment, therefore there is a discrepancy in the value of a_{BG} between measurements using a ^{4}He cryostat and dilution refrigerator. The spectra measured at 40 K are in fairly good agreement, as shown in Figure S2. The fitted values of λ and β measured at 40 K are 0.053(3) μsec^{-1} and 1.43(9) (using a ^{4}He cryostat), 0.061(2) μsec^{-1} and 1.29(5) (using a dilution refrigerator), that doesn't have a large difference.

Reference

1. Tomiyasu, K. *et al*. Conversion method of powder inelastic scattering data for one-dimensional systems. *Appl. Phys. Lett.* 94, 092502 (2009).
